# Natural variability of minimotifs in 1092 people indicates that minimotifs are targets of evolution

**DOI:** 10.1093/nar/gkv580

**Published:** 2015-06-11

**Authors:** Kenneth F. Lyon, Christy L. Strong, Steve G. Schooler, Richard J. Young, Nervik Roy, Brittany Ozar, Mark Bachmeier, Sanguthevar Rajasekaran, Martin R. Schiller

**Affiliations:** 1 Nevada Institute of Personalized Medicine and School of Life Sciences, University of Nevada Las Vegas, 4505 Maryland Parkway, Las Vegas, NV 89154-4004, USA; 2 Department of Computer Science and Engineering, University of Connecticut, Storrs, CT 06269-2155, USA

## Abstract

Since the function of a short contiguous peptide minimotif can be introduced or eliminated by a single point mutation, these functional elements may be a source of human variation and a target of selection. We analyzed the variability of ∼300 000 minimotifs in 1092 human genomes from the 1000 Genomes Project. Most minimotifs have been purified by selection, with a 94% invariance, which supports important functional roles for minimotifs. Minimotifs are generally under negative selection, possessing high genomic evolutionary rate profiling (GERP) and sitewise likelihood-ratio (SLR) scores. Some are subject to neutral drift or positive selection, similar to coding regions. Most SNPs in minimotif were common variants, but with minor allele frequencies generally <10%. This was supported by low substation rates and few newly derived minimotifs. Several minimotif alleles showed different intercontinental and regional geographic distributions, strongly suggesting a role for minimotifs in adaptive evolution. We also note that 4% of PTM minimotif sites in histone tails were common variants, which has the potential to differentially affect DNA packaging among individuals. In conclusion, minimotifs are a source of functional genetic variation in the human population; thus, they are likely to be an important target of selection and evolution.

## INTRODUCTION

Although there is a good mechanistic understanding of the processes giving rise to mutations, we know relatively little about how mutations give rise to diverse phenotypes in populations. In humans, 5–10% of known single nucleotide polymorphisms (SNPs) are thought to be in protein coding regions, which is a disproportionate share of genetic variation considering that only about 1.5% of the genome codes for proteins (1–4). Mutations in coding regions can affect protein stability and solubility, enzyme activity and many other molecular functions of proteins and nucleic acids.

Located in the coding region of proteins are minimotifs [also called short linear motifs (SLiMs)], short contiguous peptide sequences (2–15 amino acids long) with a known molecular function (5–7). Minimotifs can bind to other molecules, code for post-translational modification (PTM) sites where minimotif-containing proteins are covalently modified, and traffic a protein to a specific subcellular compartment (5). Due to its relatively short length, an SNP, or a missense mutation that changes a single minimotif residue, can diminish or eliminate a minimotif function. For example, mutation of either position of an Asn-x-Ser/Thr N-glycosylation motif would eliminate a glycosylation site (8). Likewise, a mutation can introduce a minimotif function into a site where the function did not previously exist. (Lys-x-Ser mutated to Asn-x-Ser creates a new glycosylation site.)

We therefore considered whether minimotifs are targets of selection and evolution. Several pieces of evidence support this hypothesis: (i) mutations alter minimotifs and can adversely impact human health, as exemplified by familial hypercalciuria, a disease associated with renal failure wherein a mutation of a Thr-Arg-Val minimotif mistargets Claudin 16 to lysosomes (9); (ii) viruses also use minimotifs to hijack cellular functions to facilitate their replication (10) and (iii) one of our recent collaborative studies shows that the number of tyrosine kinase signaling circuits expands in more complex multicellular organisms (11). These circuits are based upon combinations of minimotifs. If minimotifs vary among species, this would suggest that they also vary in organism populations, and if so, a subset may be under selection.

To objectively examine whether minimotifs are targeted by evolution, we examine how minimotif instances vary among humans in the 1000 Genomes Project (12, 13). In the analysis presented herein, we observe several intriguing patterns indicating that minimotifs are targets of evolution and contribute to diversity in human phenotypes. The most significant observations suggest that different people will vary in global phosphorylation states of cellular proteins and epigenetic-based cell responses to the environment. Furthermore, most minimotifs have been purified by selection, but thousands of minimotifs are variable in the human population. These results collectively support minimotifs as an element of human diversity and as a target of selection and evolution.

## MATERIALS AND METHODS

### Materials

The SNPs and reference genome were downloaded from the 1000 Genomes Project Consortium's ftp site (Version 3 of Phase 1, March 2012 release) (12,13). For SNPs, we used the Phase 1 integrated call sets and dbSNP build 132 (13,14). We used the Ensembl database to map exons to their reference genome and validated our results by aligning the resulting protein sequences to RefSeq proteins (15). All data analysis was conducted using Java, BioJava 3.0.1, MySQL, R and MySQL for R (16).

Genomic Evolutionary Rate Profiling (GERP) scores were downloaded from the UCSC genome browser(17). These constraint scores were calculated using 35 mammalian species. Constraint scores >2 were considered constrained as previously reported (17,18). Scores of exactly 0 were omitted. These GERP scores are part of the phase1 analysis of the 1000 Genomes Project were also used for some SNPs (13). PhyloP and Sitewise Likelihood Ratio (SLR) scores were obtained from dbNSFP (19,20).

Gencode3 pseudogenes were obtained from pseudogene.org (21). Our analysis included 16 000 coding sequences from 8900 pseudogenes. We also included some 350 000 transcription factor binding sites (TFBS) from the Ensembl regulation build 75 (15).

### Preparation

We used the GRCh37 reference genome and the Ensembl database to create a proteome predicted by the reference sequence for all 22 autosomes plus the X chromosome (15,22). To verify the accuracy of our mapping procedure, we aligned all protein sequences generated from the reference to known RefSeq proteins (14). We considered a predicted reference protein valid only if: (i) residues 2–15 of the query exactly matched the reference; (ii) the protein lengths were identical; and (iii) an overall identity of at least 90% was reported. Preprocessing of SNPs involved the removal of single nucleotide variants (SNVs) not present in dbSNP build 132 (14). Indels and structural variants were also ignored.

The frequency of SNPs within different categories of genomic elements was analyzed using only genomic positions of high variant call confidence (masked data). This helps avoid false positive SNP calls from G-C enriched sequences. Random sampling of 12 base segments from masked regions (pseudogenes, TFBS, minimotifs, coding regions, whole genome, minimotifs subactivities) was used for data analysis.

### Procedure and protocols

For each of the 1092 genomes, we altered the reference sequence by substituting exome SNPs for the values contained in the reference sequence. We then constructed a proteome for each person using a procedure similar to what was outlined to create the reference proteome. Alignment verification was not repeated for individual proteomes. We then created a position weight matrix of amino acids for the length of the entire reference proteome. This provides match fractions for each amino acid at each position in the reference proteome (see Equation 1).

Differences in derived allele frequencies for two sub-populations (ΔDAFs) were calculated for each loci between the continents Asia, Europe and Africa. Analysis of population differentiation excluded small populations or populations with significant admixture as previously described (13).

For each residue position of each protein analyzed, the position weight matrix provides a match fraction for each amino acid, *k*, at position *j*, where *j* < 13 × 10^6^. Each person was given a weight of 1 (so a heterozygous match for alanine for one person would be reported as 0.5). The overall match fraction for one position is the mean match fraction for the given amino acid at the given residue position. Three different representations of this matrix are given in Equation (1).

(1) \begin{equation*} \begin{array}{*{20}c} {M_{k,j} = \frac{1}{{1092}}\sum\limits_{i = 0}^{1092} {I(X_{i,j} = k = residue\;fraction\;of\;one\;person)} } \\ {\left( {\begin{array}{*{20}c} {match\;fraction_{Alanine,1} } & \cdots & {match\;fraction_{Alanine,j} } \\ \vdots & \ddots & \vdots \\ {match\;fraction_{Tryptophan,1} } & \cdots & {match\;fraction_{Tryptophan,j} } \\ \end{array}} \right)} \\ {\left( {\begin{array}{*{20}c} {Alanine,1} & \cdots & {Alanine,j} \\ \vdots & \ddots & \vdots \\ {Tryptophan,1} & \cdots & {Tryptophan,j} \\ \end{array}} \right)} \\ \end{array} \end{equation*}

### Evaluation of minimotifs

While other groups have built databases focused on select groups of minimotifs (e.g. Merops and CutDB contain protease sites), our group has built Minimotif Miner (MnM), a comprehensive database that aims to include all experimentally verified minimotif instances and consensus sequences (23–25). After new updates, MnM now contains ∼600 000 minimotifs. We include a minimotif in MnM if there is published experimental evidence for it.

We used Minimotif Miner 3 to evaluate the match fractions of amino acids occurring within minimotifs (24). For data analysis, we considered two types of minimotif mutations: (i) those that changed any residue and (ii) those that changed a critical residue. Critical residues are defined by the minimotif activity class. We consider alterations to critical residues to be potential loss-of-function minimotif (LOFM) variants (see Table 3). In the special case of phosphorylation residues, we recognize that some modifications result in changes between serine and threonine. Such modifications were not considered LOFMs and were reported separately.

**Table 1.** Summary of minimotifs with SNPs

Activity	Minimotif Critical Residue SNPs		Missense minimotifs		Minimotifs with SNPs		Total
	*N*	%	*N*	%	*N*	%	*N*
Acetylation	75	0.396	1214	6.33	2601	13.6	**19 179**
Di-methylation	10	0.907	73	6.62	172	15.6	**1103**
Glycosylation	18	0.413	252	5.79	502	11.5	**4356**
Lipid	1	0.429	14	6.01	26	11.2	**233**
Mono-methylation	17	1.083	108	6.88	240	15.3	**1569**
O-glcnac	1	0.424	12	5.08	32	13.6	**236**
Phosphorylation	900/84^a^	0.498	13 751	7.6	27 613	15.3	**180 891**
Proteolysis	140	0.49	1389	4.86	3296	11.5	**28 571**
Tri-methylation	1	0.571	17	9.71	40	22.9	**175**
Ubiquitination	79	0.275	1679	5.83	4017	14	**28 775**
Sumoylation	0	0	27	3.49	86	11.1	**773**
Dephosphorylation	0	0	6	2.46	29	11.9	**244**
Nitration	0	0	0	0	3	21.4	**14**
**Total**	**1243**	0.467	**18 542**	6.97	**38 657**	14.5	**266 119**
**Distinct SNPs**	**1044**	**-**	**10 961**	**-**	**22 846**	**-**	**-**

^a^We observed 84 polymorphisms encoding Ser-Thr substitutions in minimotifs in addition to the 900 phosphorylation site SNPs.

**Table 2.** Summary of phosphorylation minimotifs

Type of motif alteration	SNPs	Minimotifs	%
Derived minimotifs	43	48	0.025
Serine–threonine transitions	67	84	0.046
Critical residue mutations	757	900	0.50
Non-synonymous SNPs	8273	13 751	7.6
Minimotifs with SNPs	16 893	27 613	15.3

**Table 3.** Summary of DM and LOFM minimotifs

^a^Summary of minimotif SNPs	DMs	LOFMs
Acetylation	2	67
Di-methylation	0	10
Glycosylation	0	14
Lipid	0	1
Mono-methylation	0	14
O-glcnac	0	1
Phosphorylation	48	765
Proteolysis	3	112
Tri-methylation	0	0
Ubiquitination	3	71
**Total**	**56**	**1055**

^a^Limited to minimotifs with autosomal SNPs where the ancestral allele is known.

We used a MySQL interface for R and Excel to create plots for figures. Boxplots of GERP scores were created by selecting distinct SNPs with GERP scores for each activity class. Comparison of GERP scores across proteins, SNPs, and minimotif SNPs only considered distinct SNPs with GERP scores that fell on exome regions passing validation.

## RESULTS

### Minimotifs are polymorphic in the human population

Since some minimotif-based signaling circuits evolutionarily expanded in more complex multicellular organisms (11), we hypothesize that minimotifs might be polymorphic in organism populations and provide a basis for selection of signaling, trafficking, and regulatory networks. The recent completion of a sequence of 1092 genomes in the 1000 Genomes Project let us test this hypothesis (13,12,26). First, we asked how many minimotif instances are polymorphic in the human population. In proteins, minimotifs are short peptide regions associated with a known molecular function. The Minimotif Miner 3 (MnM) database has ∼300 000 minimotifs (24). To update this database, we re-ran parsers to import data from other databases. Our combined new version contained ∼600 000 minimotifs, of which 300 332 were for human proteins. Using a MySQL query, we extracted the set of minimotif instances with a known position in a human RefSeq protein. Then we wrote a custom Java program mapping the RefSeq protein sequence to the Ghr37.3 reference human genome.

For each of the 1092 genomes in the phase 1 call sets of the 1000 Genomes Project, we used coding SNPs to predict the variability in peptide sequence for ∼23,000 RefSeq proteins (12,13). We then mapped the minimotifs from the MnM database to nucleotide positions in the Ghr37.3 and calculated the frequencies of missense variants in minimotif instances for the 1092 genomes. To ensure the accuracy of our program, we compared the SNP calls with data in the dbSNP database and verified that almost all were previously known SNPs. This analysis was performed for experimentally verified minimotif instances and not for minimotif predictions from consensus sequences.

**Figure 1.**
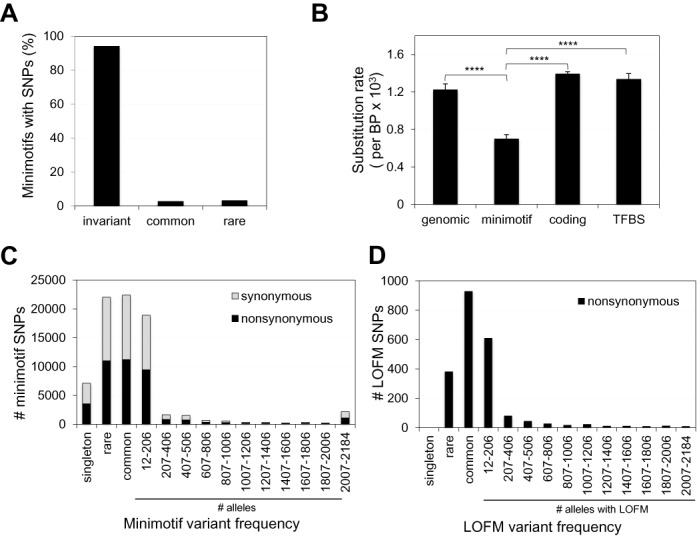
Minimotifs show functional constraint. (**A**) Histogram of minimotif conservation. (**B**) Histogram of substitution rates for different genomic regions including minimotifs [*n* = 5 (50 000, 12 bp segments)]. **** indicates *P* < 0.0001 for differences in pairwise comparison to minimotifs SNPs. (**C**) Stacked Histogram for occurrences of variant SNPs located in minimotifs. Synonymous and nonsynonymous SNPs are shown. (**D**) Histogram for SNPs that alter the amino acid of a residue that is post-translationally modified (LOFMs). (**C** and **D**). The rare variant fraction has a MAF < 1%.

We predicted the amino acid substitutions encoded by 455 336 SNPs within the coding sequences of 22 762 human RefSeq proteins. Approximately 282 000 minimotif instances have the same sequence as the reference genome and are fixed in the population (Figure 1A). This high conservation suggests that the vast majority of minimotifs (94%) were purified from ancestors by selection. To compare the conservation of minimotifs to other genomic regions we randomly sampled 50 000 12 nt regions five times for minimotif instances, all genomic SNPs, coding regions, and TFBSs, and calculated the substitution rate per base pair. The substitution rates for minimotifs were significantly lower than those for genomic SNPs, coding regions, and TFBSs (Figure 1B). We also note that while ∼5% of all coding SNPs occur in minimotifs, about 15% of missense mutations occur in minimotifs, indicating that minimotifs are mutated, on average, more often than the rest of the protein. We conclude that the majority of minimotifs are more constrained than TFBSs, which are similar in size. We were surprised that minimotifs were also significantly more constrained than coding regions in general, which could be explained by the fact that a single point mutation in a minimotif instance is likely to cause loss of function, whereas this is generally not true for most positions in coding regions.

There are similar levels of common and rare variants in minimotif instances. Of the polymorphic minimotifs, 9548 (3.2%) had rare allele frequencies <1.0% (Figure 1A). Singletons comprised 2807 of the SNPs in minimotifs, occurring just once in the 1092-person cohort (Figure 1C); a small fraction of these variants may reflect sequencing errors. 8055 SNPs in minimotifs (2.7%) were common variants, similar to the percentage previously reported for TFBSs (27). The minimotif allele frequency distribution showed a strong preference for minor allele frequencies (MAFs) of <10%. Given the large numbers of minimotif instances, the number of possible permutations of those containing common and rare variants, and the frequencies of these variants (Figure 1C), there are likely many different minimotif haplogroups in the human population.

### Loss of function minimotifs

Missense SNPs that mapped to degenerate positions within minimotif instances could potentially encode substitutions that do not impact the function of the minimotif. For example, a mutation in a degenerate ‘x’ position of the PxxP minimotif that binds an SH3 domain would likely have little effect on binding. Therefore, we wanted to determine polymorphisms in minimotifs with LOFMs. The Minimotif Miner database data model includes an ‘mmod’ field containing the amino acid position that is covalently modified by a PTM, as well as the type of PTM (5). We used a SQL query to identify the subset of minimotif instances that was both variable in the human population and also contained a mutation for the mmod residue position that is covalently modified. These mutations most certainly represent LOFMs, except in rare cases of substitution of a functionally equivalent residue. For example, if a phosphorylated Ser residue in a minimotif is mutated, the mutation is unlikely to be functional. There is an exception where some kinases with dual specificity can phosphorylate both Ser and Thr; in this case, the Thr substitution would be functional.

This analysis revealed that 1055 (4.6%) of all minimotif instances with an SNP in the 1092 genomes were LOFMs. Approximately 0.4% of all ∼300 000 minimotifs were LOFMs, which is comparable with that of loss-of-function mutation in mRNA splicing sites (0.15%; 707 of 233 785 exons with two splice sites per exon)(28). The percentage of minimotifs LOFMs was higher than that of nonsense mutations that are likely to be loss-of-function (0. 01–0.3%); see MacArthur et al. or estimated from 1296 of ∼10 million amino acid position (13,29).

The activities of LOFMs were mostly for phosphorylation sites (*n* = 900; Table 1). However, other activities included mono-, di- and tri-methylation, acetylation, ubiquitination, and proteolytic cleavage sites. Of the LOFM substitutions, 555 were observed in at least 1% of the population. There were 382 rare SNPs that were LOFMs, no singletons, and the majority were low frequency common variants (MAF < 10%; Figure 1D). If we consider the combinatorial complexity of these LOFM sites alone, there are many times more permutations than people who have ever lived. Collectively, we conclude that genomic variability in minimotif coding regions is a source of molecular functional diversity in the human population.

### Selection of minimotifs

**Figure 2.**
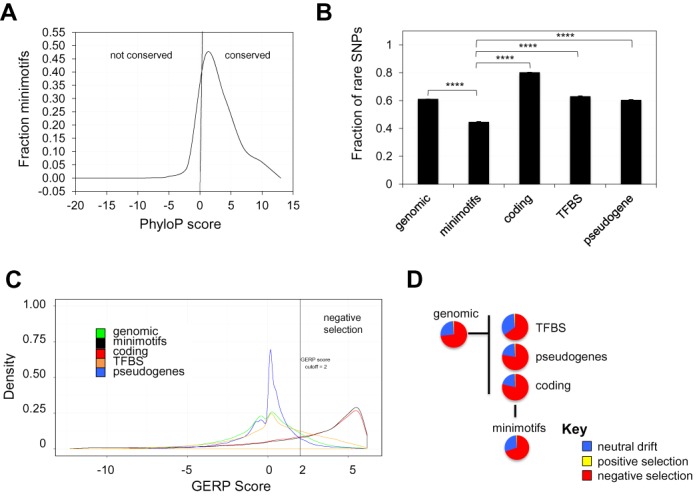
Approximately half of nonsynonymous SNPs for minimotifs have undergone purifying selection. (**A**) Minimotif conservation plot showing PhyloP scores. (**B**) Histogram of fraction of rare SNPs for different genomic regions including minimotifs [*n* = 5 (50 000, 12 bp segments)]. **** indicates *P* < 0.0001 for differences in pairwise comparison to minimotifs SNPs. (**C**) Density plots showing the distribution of GERP scores for different genomic regions (see legend). A vertical line indicates the GERP score threshold of 2. (**D**) Pie graphs showing fraction of genomic elements with types of selection based on the SLR statistic for different genomic regions; see key, SLR < 4 = negative selection, 4 ≤ SLR ≥ 6 = neutral drift, and SLR > 6 = positive selection.

To assess how well minimotif instances are conserved among species, we examined conservation using PhyloP scores for conservation from the dbNSFP database, which measures conservation among 100 vertebrates including humans (20). The more positive the score, the more highly conserved the site (Figure 2A). Most minimotif instances have scores above 1 (median = 0.9), indicating that most minimotifs are somewhat conserved among vertebrates. However, a significant fraction of minimotif instances have low or negative scores, suggesting they may be more recently acquired in the vertebrate lineage.

While only a small percentage of minimotifs (6%) are variable in the 1000 Genomes cohort, a substantial number of minimotifs contain variable alleles (Table 1), indicating that minimotifs are a source of human variability at the genomic level. Therefore, we hypothesize that minimotifs are targets of selection. To test this hypothesis we compared the fraction of rare SNPs in minimotif instances to several other genomic regions. This fraction is considered a metric for negative selection where a high fraction of rare variants indicates that the regions are under negative selective pressure (30).

The fraction of rare variants in minimotif instances was 0.49, significantly lower than that for all genomic regions, coding regions, and TFBSs, and even lower than that for pseudogenes (*P* < 0.0001); the previously reported rare variant fractions for coding regions, pseudogenes, and TFBSs were similar to our calculated values (30) (Figure 2B). Furthermore, the fraction of rare minimotif SNPs was also lower than that reported for UTRs, enhancers, noncoding RNAs and other noncoding regions (30). This result suggests that the portion of minimotifs that are variable are under less negative selective pressure than most other genomic regions (Figure 2B). The fraction of rare LOFMs (0.31) was lower than all minimotifs and pseudogenes, indicating that variants in critical minimotif residues are mostly for common variants, although they generally have low MAFs of less than 10%.

To further investigate whether the variable human minimotifs are under purifying selection in vertebrates, we used Genomic Evolutionary Rate Profiling (GERP) scores (17, 18, 31). GERP scores for this subset ranged from −12.3 to 6.2, with 6.2 being the strongest indicator of purifying selection. A density and box plot (Figure 2C, Supplementary Figure S1) shows that the median minimotif GERP score is ∼2, similar to coding regions that also share similar GERP score density plot distributions; a similar average GERP score was previously reported for coding regions (18). A GERP score threshold of 2 was previously used as a threshold for indicating purifying selection (17, 18). This plot indicates that minimotif instances, like coding regions, are under strong restraint, unlike genomic SNPs, pseudogenes, and TFBSs (Figure 2C); introns and UTRs also have low average GERP scores supporting strong restraint (18).

To verify negative selection and test for positive selection, we used the SLR statistic (33). We were concerned that the thresholds selected for the SLR statistic might drastically affect conclusions about negative selection, positive selection, and neutral drift. (Note that a value of one is neutrality.) We examined SLR density plots for natural break points and observed such a break point at a value of -3 (Supplementary Figure S2), which was then used as a threshold to segregate negative selection and neutrality. For distinguishing neutral drift from positive selection, we used a value of +3, where the percentage of coding, minimotifs, and pseudogenes started to show some divergence. We consider the neutral drift category to contain minimotif variants under neutral drift and possibly also under weak selection. The percentage of each selection type was compared using less conservative thresholds for neutrality (−6 > SLR < 4), producing similar results that did not alter our overall conclusions. Furthermore, the likelihood ratio test (*ω*) values were generally less than one supporting negative selection (data not shown)(34).

We compared SLR scores for minimotif SNPs to those for all genomic SNPs and SNPs in TFBSs, pseudogenes, and coding regions. Results for the SLR analysis were generally consistent with the conclusions from the rare variant fraction and GERP score analyses. For most categories, only 1–2% of genomic elements had SLR scores suggesting positive selection (Figure 2D). The largest changes were in the ratio of SNPs under neutral drift or weak selection (−3 > SLR < 3) to those under negative selection, where each category differed from all genomic SNPs. We found that 73% of minimotif instances were under negative selection, with the majority of the remainder under weak or no selection. TFBSs are similar in size to minimotifs, and were similarly constrained, with 68% under purifying selection. Coding regions and pseudogenes were the most constrained, with 78–79% of SNPs showing the SLR signature of negative selection. The negative selection of pseudogenes based on the SLR statistic was unexpected, as our GERP score analysis of pseudogenes was not consistent with negative selection and previous studies supported neutral evolution of most pseudogenes (35,36). This point is addressed in the Discussion section.

### Selection of minimotifs activity types

**Figure 3.**
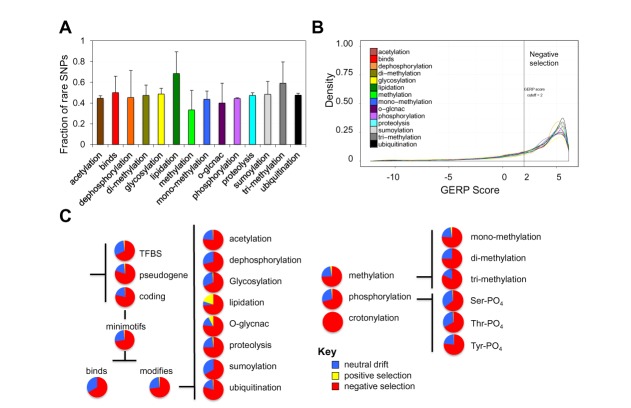
Some minimotif activities are more prone to selection than others. (**A**) Histogram of fraction of rare SNPs for different types of minimotifs. (**B**) Density plot showing the distribution of GERP scores (for different minimotif subactivities (see legends). A vertical line indicates the GERP score threshold of 2 (B). (**C**) Pie graphs showing the percentages of SNPs in different genomic regions and activity subgroups under neutral, negative, and positive selection as measured by the SLR statistic in Figure 2. Lipidation, O-glcnac, dephosphoryation and crotnylation had small numbers of minimotifs (*n* < 20).

We investigated whether different types of minimotifs had varying selective pressures using rare variant fractions, GERP scores, and the SLR statistic. There were 16 different types of minimotifs present in the Minimotif Miner Database that were analyzed. We did not look at trafficking minimotifs because there were so few. The rare variant fraction analysis showed values from 0.3–0.7, with tri-methylation and lipidation minimotifs having the highest fraction of rare SNPs (Figure 3A). However, while trends were observed, no minimotif activities significantly differed from one another.

We examined density plots and quartile plots for GERP scores segregated by different minimotif activities (Figure 3B, Supplementary Figure S3). Minimotifs have differing binding or PTM activities. Most minimotifs subactivities had GERP scores >2, indicating purifying selection consistent with the negative selection and interspecies conservation of minimotif instances (Figure 2). Glycosylation minimotif activities had generally low GERP scores (Figure 3B, Supplementary Figure S3), suggesting that most glycosylation sites may not be under strong purifying selection. However, glycosylation sites are necessarily positionally conserved, which would not be detected in our calculations.

As a separate confirmatory approach, we examined the SLR statistic for different minimotif types. The major types of minimotifs for binding and PTM sites had similar levels of selection to minimotifs with most under purifying selection and a ∼30% portion under neutral drift or weak selection (Figure 3C, Supplementary Figure S4). Likewise, most minimotif activities had similar proportions under purifying selection and neutral drift. Ubiquitination and tri-methylation had less neutral drift and crotonylation sites were under negative selection, although this was only for six sites. Lysine crotonylation is a type of histone PTM that activates promoters similar to acetylation (37). As noted in Figure 2D, very few minimotif instances were under positive selection; lipidation and O-glcnac modification subactivities had 8–20% instances under positive selection.

**Figure 4.**
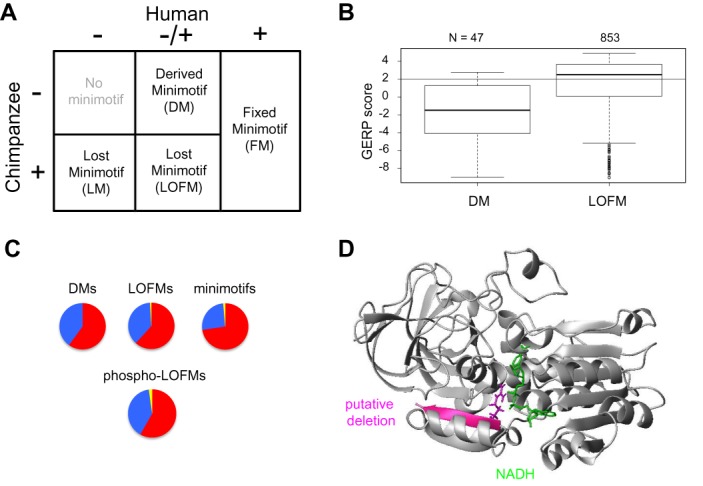
Most loss of function minimotifs have undergone purifying selection and most newly derived minimotifs have not. (**A**) Grid showing naming convention for SNPs that alter the modified residue of a minimotif and the frequency of alleles in humans and chimpanzees. (**B**) Quartile boxplots for GERP scores for LOFMs and DMs. The number of SNPs for each category is shown on top. A horizontal line indicates the GERP score threshold of 2. (**C**) Pie graphs showing the percentages of SNPs minimotif groups under neutral, negative, and positive selection as measured by the SLR statistic in Figure 2. (**D**) Ribbon diagram of the structure of the Alcohol Dehydrogenase 1B protein (1HSZ) showing the NADH cofactor (green), a β–strand for the last 5 amino acids in the protein (magneta) and the polymorphic amino acid involved in alcoholism (R370, magenta)(89).

We investigated the gain and loss of minimotif functions in humans. The vast majority of minimotifs (94%) are invariant in humans and designated ‘Fixed Minimotifs’ (FMs; Figures 1A and 4A). The remainder of minimotif instances were polymorphic in the cohort. Derived Allele Frequencies (DAF)s measure how quickly alleles that are new to humans, as opposed to other primates, are approaching fixation in the human population. We use the term Derived Minimotif alleles (DMs) to describe those minimotifs with SNPs that alter an amino acid in a PTM site and are not present in chimpanzees. As expected, the majority of DMs had GERP scores below two, which was supported by SLR analysis with approximately half the minimotifs under negative selection (Figure 4B and C). Most of these DMs were for phosphorylation sites (Table 2).

Most LOFMs, as defined previously, had GERP scores that were >2 and greater than 60% had SLR scores that suggested purifying selection (Figure 4B and C) (38). Most of these minimotifs were present in a common primate ancestor, but a subset had variants in different individuals of the human population. The majority of these LOFMs were for phosphorylation sites, although a significant number of acetylation, ubiquitination, and proteolysis sites were also observed (Table 1). These results would be expected if new minimotifs were acquired in humans and these alleles were actively spreading in the human population. Lost minimotifs (LMs), those present in chimpanzees but not observed in humans, could not be assessed given available data.

Since ∼70% of minimotifs in the MnM database are for phosphorylation sites, we examined these sites alone. Approximately 15% of the phosphorylation minimotif instances had an SNP, of which half were non-synonymous (Table 2). A higher proportion (0.5%) of all phosphorylation sites had LOFM mutations. Indeed, ∼0.05% had a possible functional transition between Ser and Thr at the phosphorylation sites.

### Minimotif case studies

We identified ∼94% of human minimotifs as purified by selection. This implies that they serve important functions, and that mutation of DNA encoding minimotifs should be associated with disease. However, our 2007 review of minimotifs in disease revealed that few minimotifs are involved (10,39). This discordance suggests that many minimotifs involved in disease mechanisms remain to be discovered. Therefore, we examined whether any of the LOFMs could impact health. For each LOFM, we analyzed whether its source protein was associated with a disease as annotated in the OMIM database (40). This was cross-referenced through the Human Reference Protein Database (HPRD), an encyclopedia of human proteins (41). We examined the literature to determine if any minimotif-altering SNPs could further our knowledge about human disease. We identified the previously reported K751Q polymorphism (rs13181) that eliminates an ubiquitination site in the *ERCC2* protein and is associated with greater risk of melanoma, acute myeloid leukemia, and Xeroderma Pigmentosum (42,43). Two other case studies, described herein, show the value of this type of analysis.

One of the major alleles associated with alcoholism (rs2066702) results in a R370C mutation in Alcohol Dehydrogenase 1B, which is under positive selection in the human population (44,45). Analysis of mice null for *ADH1B* show that this gene plays a part in the detoxification of acute exposure to alcohol (46). Individuals with this mutation do not metabolize alcohol well and the mutation results in a 70-fold decrease in the affinity for NAD^+^ (47). Since R370 makes contact with the NAD^+^ cofactor in crystallographic studies (Figure 4D), it is inferred that this interaction is responsible for the low *K*_m_. However, our minimotif analysis has identified a proteolytic site introduced by a missense mutation encoding R370C that could affect the stability of the C-terminal fold, and could result in cleavage of the last five amino acids off the ADH1B protein. This cleavage would not be observed as a change in molecular mass on Western blots (47), but should drastically reduce enzyme activity, as these residues are part of the active site.

Another example of a LOFM that may impact human health, SNP rs3750050, is associated with an increased risk for colon cancer (48). This allele results in a T573A mutation in the *PTPN12* protein. *PTPN12* encodes a non-receptor protein tyrosine phosphatase and the T573A mutation decreases its phosphatase activity (48). Our minimotif analysis identified T573 as a site of phosphorylation (49), providing a link and potential mechanism for how this mutation alters the phosphatase activity.

### Minimotifs in histones

Several minimotif sites were for PTMs commonly found in N-terminal histone tails, which play a role in nucleosome assembly/disassembly, chromatin accessibility, epigenetics and genomic imprinting. Thus, polymorphic histone minimotifs could potentially influence epigenetics.

**Figure 5.**
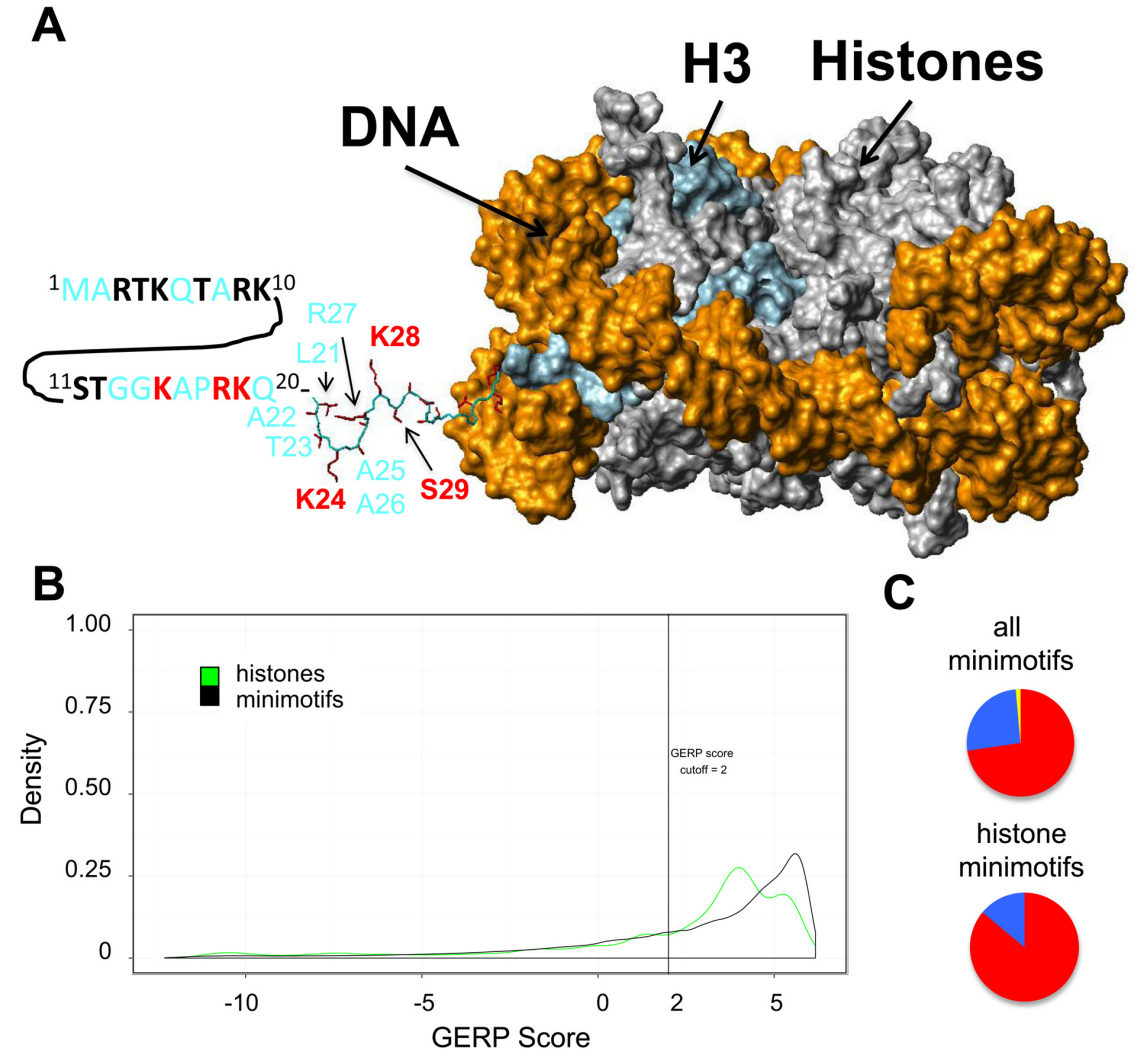
SNPs present in histone minimotifs. (**A**) Surface plot of nucleosome showing locations of polymorphic minimotif residues. Histone H3 is colored light blue and DNA is colored gold. The structure is from 1AOI and accession number for the sequence is P02302. A portion of the N terminal tail of histone H3 that resolved in the crystal structure is shown as a stick figure with side chains colored red. The sequence of the portion of the N-terminal tail that did not resolve (residues 1–20) is shown as a sequence using single letter amino acid code. Labels in black or red bold font indicate sites of covalent modification in minimotifs. Fonts colored black are conserved and those colored red are polymorphic. Fonts colored cyan are neither polymorphic nor post-translationally modified. No polymorphic residues were located inside the core of histone H3. (**B**) Density plots for GERP scores for minimotifs present in the 53 human histones. A vertical line indicates the GERP score threshold of 2. (**C**) Pie graphs showing the percentages of histone minimotif SNPs groups under neutral, negative and positive selection as measured by the SLR statistic in Figure 2.

The MnM3 database contains a total of 933 different modifications (eight different activity types) for the 53 human histone genes. Histones have many minimotifs with an average of 26 minimotif instances per histone gene and a range of 1–113 minimotifs for the 53 histone genes. Most histone minimotif instances (*n* = 895) were invariant in the sample population; however, 38 histone minimotifs were >1% polymorphic and five of the SNPs encoded LOFMs in histones. Figure 5A shows the example of histone H3 with the locations of minimotifs with SNP in the N-terminal tail. Histone minimotifs tended to have higher GERP scores than the complete set of all minimotifs (Figure 5B). Approximately half of histone minimotif instances with SNPs had GERP scores >2 and a higher percentage were under purifying selection. This was supported by the SLR statistic (Figure 5C) Considering this subset and the invariant histone minimotifs, most have been purified or are under purifying selection. However, there is a subset of polymorphic minimotifs in histone tails that are a source of variation and could affect differences in individual response to the environment.

### Minimotifs show a variable geographic distribution

**Figure 6.**
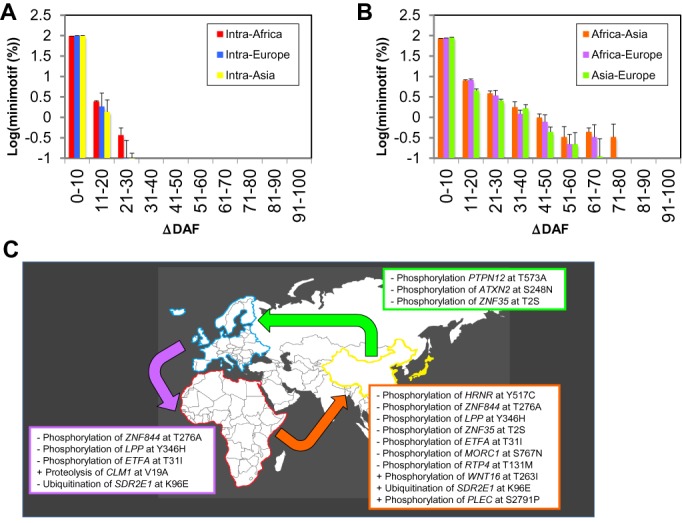
Minimotif alleles have a variable geographic distribution. Bin plot showing the intracontinental (**A**) and intercontinental (**B**) variability of DM and LOFM minimotif alleles. Continents are colored with primary colors and intercontinental relationships with related secondary colors. (**A** and **B**) Standard deviations were calculate from populations groups for the continents indicated (African – ASW, YRI, LWK; European – CEU, FIN, GBR, TSI; Asian – CHB, CHS, JPT). Abbreviations for populations are from the 1000 Genomes Project (13). (**C**) A portion of the world atlas showing different minimotifs with intercontinental ΔDAFs > 50%. The embedded tables show the minimotifs, genes that contain the minimotif, minimotif functions and amino acid changes encoded by missense mutation alleles that have the highest differences between continental populations. Table rows that start with a ‘+’ indicate a DM and those with a ‘–’ indicate a LOFM. Continent and table colors are as in (**A**) and (**B**).

To identify minimotifs possibly involved in adaptation of human populations to different niches, we examined ΔDAF scores. ΔDAF is a change in the frequency of derived alleles between two human subpopulations. We limited this analysis to DMs and LOFMs (*n* = 1029). We looked at the variability of these minimotifs among different ethnic groups among and between continents, geographic groups previously examined in the 1000 Genomes Project (13). Within continents, a low variability for these minimotifs was observed, with no minimotifs having pairwise ΔDAF scores >21% (Figure 6A). There was slightly more variability between African groups, as expected based upon early segregation of these groups in human history (13). When we examined the intercontinental variability in minimotifs, ΔDAF were much higher, with some minimotif alleles reaching as high as an 80% difference in DAF scores (Figure 6B). Notably, no minimotifs were completely fixed in one population and under selection in another. If minimotifs were under selective pressure, we would expect a spread pattern with more intercontinental variability among minimotifs alleles than among regional populations as observed here.

Since some minimotifs had very high intercontinental ΔDAFs, we can identify those minimotifs under differential selective pressure in the human population. The 13 minimotifs with high ΔDAFs above 50% help to identify not only those genes under selective pressure, but molecular minimotif functions. Minimotifs with high pairwise ΔDAF are shown in Figure 6C. Several of the genes are related to interaction with the environment and reproduction, including transcription factors, skin keratinization and wound healing, spermatogenesis, mitochondrial function and immune function. Most of the molecular functions were for phosphorylation sites and were LOFMs. Several were more common to both Europeans and Asians, when compared to Africans. Many other minimotifs had high ΔDAFs, although not as high as those shown in Figure 6C (Supplemental Table S1). Thus, our minimotif analysis provides insight into a large set of molecular functions that, based on geographical distribution, appear to be currently under selection in the human population.

## DISCUSSION

The ability to rapidly sequence human genomes and exomes has allowed us to explore the variability in sequence and the encoded structure and function among individuals. Notable variability has been observed in gene copy number, transcription factor binding sites, mRNA splicing sites and DNA methylation (50,51). Several studies have predicted the effects of nonsynonymous SNPs on protein function. The majority assess effects upon the function of the gene, the molecular pathways of the gene, the structure or biophysical characteristics of the protein, pre-mRNA splice sites or the phenotypes (52–66). For example, Polyphen, SIFT and SNPeffect provide predictions for effects of SNP on protein functions and structures (67,61,68,55). Predictions have limitations, however, as evidenced by a benchmark study of four different SNP-function predictors where only 11% of the predictions were consistent among the applications (69).

### Selection of minimotifs

Other studies have suggested that minimotifs are targeted by evolution, but this has not been rigorously tested at the population and genomic levels (70–73,42,74,75). We thought that minimotifs could be targets of evolution because a single missense point mutation can generate a new derived minimotif, or alternatively create a loss of function for an existing minimotif. Given that our MnM database has ∼300 000 minimotifs in the human proteome, minimotif annotations provide a wide diversity of molecular functions that might be variable in the human population.

Several of our findings support minimotifs as a target of evolution. There are two distinct populations of minimotifs in the human genome. One is highly invariant with completely conservation of 94% of the ∼300 000 human minimotifs previously purified by selection. A portion of the previously identified millions of conserved small islands in the genome may be minimotifs (76). A second population is variable, with relatively low substitution rates (with the vast majority of these having MAFs of <10%) yet a higher proportion of common elements when compared to most, if not all other genomic elements tested so far (30). We have focused on this subset.

We investigated selection in this second population of minimotifs. For assessing previous selection, we examined interspecies selection and conservation metrics. The minimotif analysis of GERP scores suggests that most minimotifs are under purifying selection (Figure 2C). This is supported by the positive PhyloP scores of most minimotifs, reflecting conservation among vertebrates (Figure 2A).

Several metrics were used to assess the role of selection in adaptive evolution within humans. SLR analysis supports that most minimotifs are under negative selection with ∼27% under weak selection or neutral drift. However, the rare variant fraction for minimotifs was lower when compared to any other genomic element. This pattern is supported by a significantly lower substitution rate and a very low percentage of newly derived minimotif alleles (only 47 total). The accepted interpretation of the low rare variant fraction would be that minimotifs are not under negative selection, which is in discordance with the other selection metrics we tested. Furthermore, the low MAF for most minimotifs implies that these alleles may have been recently introduced into the human population, but this is not supported by postive PhyloP scores supporting conservation.

An explanation for the discordant observations is that minimotifs fit a peculiar selective niche. Even though minimotifs have a higher fraction of common variants, the MAF for the vast majority of minimotifs is <10%. Therefore, we favor the hypothesis that most variable minimotifs are under negative selective pressure, but favor low frequency common variants. Minimotifs are often found in groups in proteins such as multiple phosphorylation sites or multiple PxxP sequences for SH3 binding sites (77). In this situation, one of the minimotifs can be mutated and result in a tolerated loss-of-function, as long as the majority of the other minimotifs in that protein are conserved. This situation has likely evolved to maintain connectivity in the network, but at the same time provide robustness for that connectivity. This could explain why we observe negative selection of common variants with low MAFs. This could also give rise to minimotif haplogroup coadaptation in adaptive evolution. The coevolution of minimotif hypothesis would need to be further tested.

Very few minimotifs were under positive selection as is generally observed for the entire genome (*n* = 214; 18 of were also LOFMs). However, protein modification and signal transduction Gene Ontology (GO) terms, enriched in positively selected sites, had the highest *P* values (78). Most sites were for phosphorylation (*n* = 164) and the remainder reflected many different types of minimotif activities. Five genes had three or more minimotifs under positive selection. Many of the minimotifs were in genes associated with human disease and we observed some specific processes, such as DNA repair and neuronal development that were well represented in the positively selected minimotifs.

Of the ∼170 000 phosphorylation site minimotifs, there were only 41 derived alleles. This is similar to the 37 newly derived phosphorylation sites previously reported (73). This suggests that the phosphoproteome network is generally stable in primates.

### Minimotifs in epigenetics

Additional support consistent with minimotifs as evolutionary targets comes from our analysis of histone minimotifs. The N-termini of histones are accessible in nucleosomes, and several different types of minimotifs are part of the histone code. These minimotifs affect DNA packaging and accessibility to the DNA, and normal and disease epigenetic phenotypes (79–82). DNA methylation was previously identified as a source of human variation (51). Although most histone minimotifs are invariant, 38 are polymorphic in the human population and 5 are LOFMs. We therefore conclude that there are 20^38^ possible haplogroups of histone minimotifs. Since many of these histone minimotifs are involved in DNA packaging, some haplogroups could vary in DNA packaging and gene expression; however, this hypothesis will require experimental testing.

We have observed several patterns of minimotif diversity in the human population, but we must consider that there are likely many more minimotifs yet to be discovered (83). The minimotifs in the MnM database used for this analysis are present in ∼21 000 of the 70 000 human RefSeq records, and only 9788 distinct protein sequences with minimotifs had any SNPs included in our study. As new minimotifs are discovered, we expect to find many more that are polymorphic or rare variants. Sequencing of human genomes to identify new epitypes at the population level is only a few years old, and sequence data is growing faster than Moore's law (84). Furthermore, the Minimotifs Miner database has been rapidly growing (6,24,85).

### Pseudogenes in adaptive evolution

We analyzed pseudogenes as a control for a genomic element generally not under constraint based on previous studies (35,36). Our GERP score analysis agreed with this previous conclusion of neutral genetic drift over a long time scale. However, our SLR analysis suggests that most pseudogenes are under negative selection in evolutionary adaptation. Our source for the SLR statistic scores was the dbNSFP database (20). There are two likely explanations for this discrepancy: (i) the previous conclusions could differ because of a sampling error. Previous studies only looked at ∼200 pseudogenes from a small section of the genome, whereas our analysis here examined ∼8900 pseudogenes from the Gencode database (86); (ii) The observed constraint on pseudogenes for a shorter time scale may reflect newly identified functions for pseudogenes in esiRNAs, small interfering RNAs and decoys for microRNAs (87,36,88). Thus, it appears that selection of pseudogenes should be revisited.

## SUPPLEMENTARY DATA

Supplementary Data are available at NAR Online.

SUPPLEMENTARY DATA
