# Supplementary material for: Natural variability of minimotifs in 1092 people indicates that minimotifs are targets of evolution
Source: Nucleic Acids Res. 2015 Jun 11;43(13):6399–412. doi: 10.1093/nar/gkv580 (PMC4513861; doi:10.1093/nar/gkv580)
Supplement: SUPPLEMENTARY DATA [file supp_43_13_6399__index.html]

Natural variability of minimotifs in 1092 people indicates that minimotifs are targets of evolution — Natural variability of minimotifs in 1092 people indicates that minimotifs are targets of evolution — SUPPLEMENTARY DATA 

# Natural variability of minimotifs in 1092 people indicates that minimotifs are targets of evolution

## SUPPLEMENTARY DATA

- SUPPLEMENTARY DATA
- SUPPLEMENTARY DATA
