# Supplementary material for: Natural variability of minimotifs in 1092 people indicates that minimotifs are targets of evolution
Source: Nucleic Acids Res. 2015 Jun 11;43(13):6399–412. doi: 10.1093/nar/gkv580 (PMC4513861; doi:10.1093/nar/gkv580)
Supplement: SUPPLEMENTARY DATA [file supp_gkv580_nar-01136-n-2015-File003.pdf]

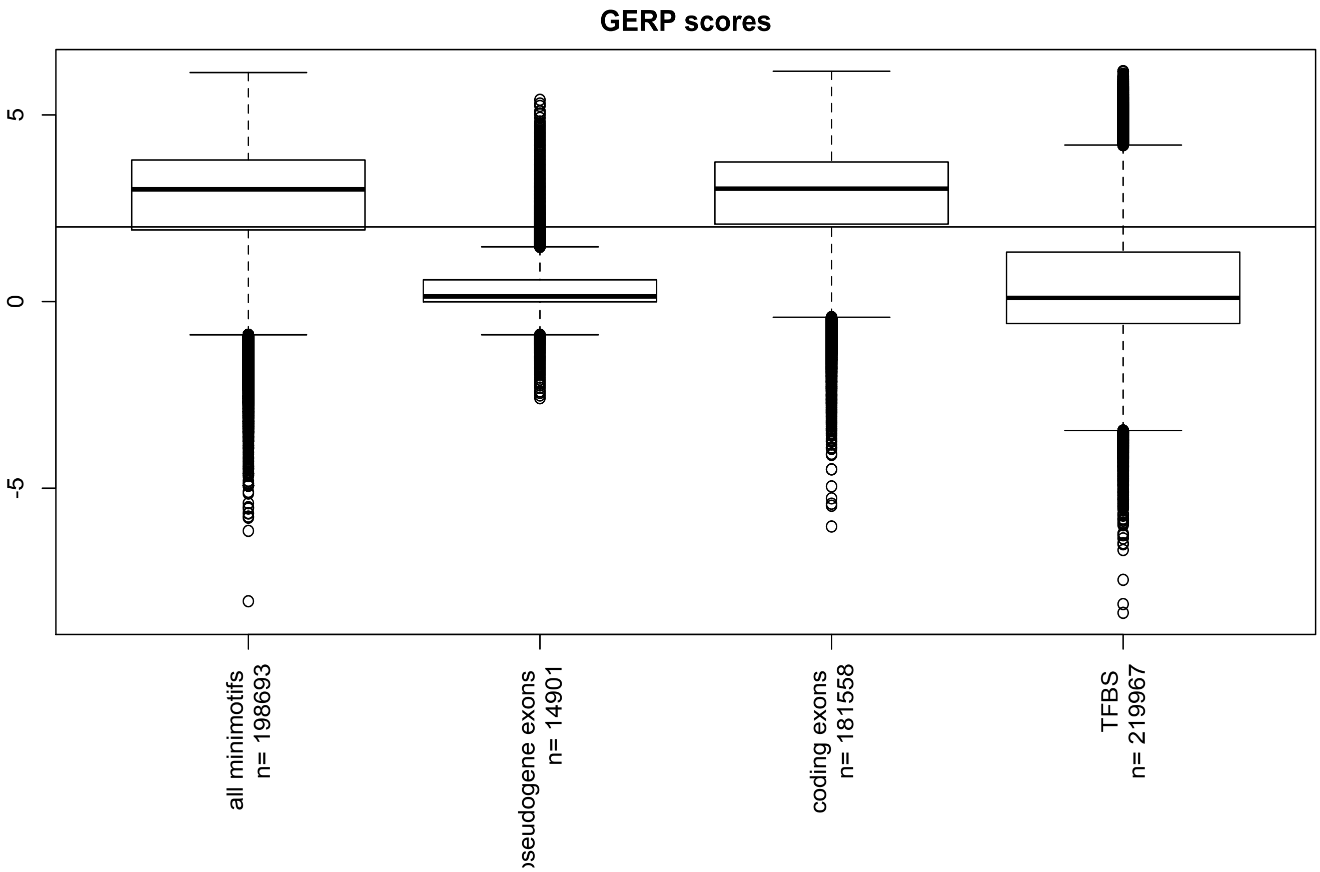

Figure S1. Quartile plots of GERP scores for different genomic regions.

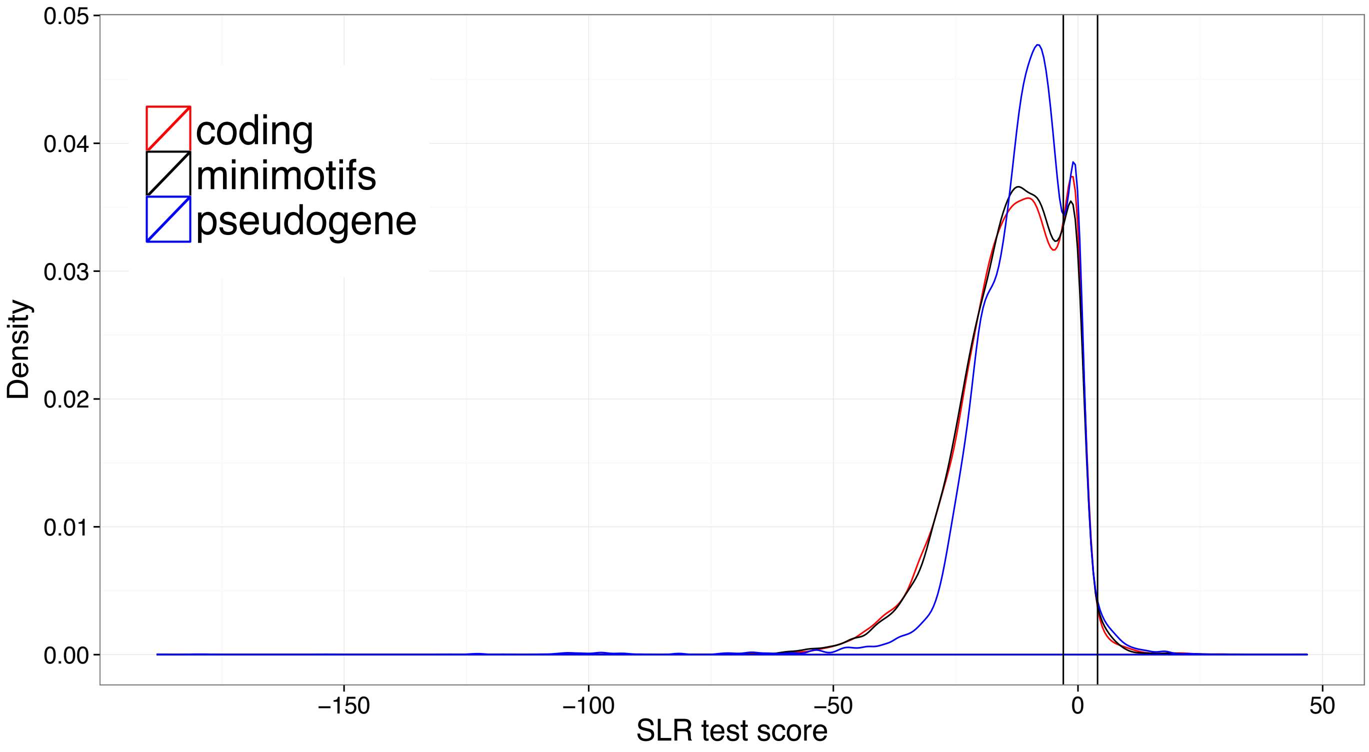

Figure S2. Density plot of SLR scores for different genomic regions. Vertical lines indicated threshold for negative, neutral, and positive selection.

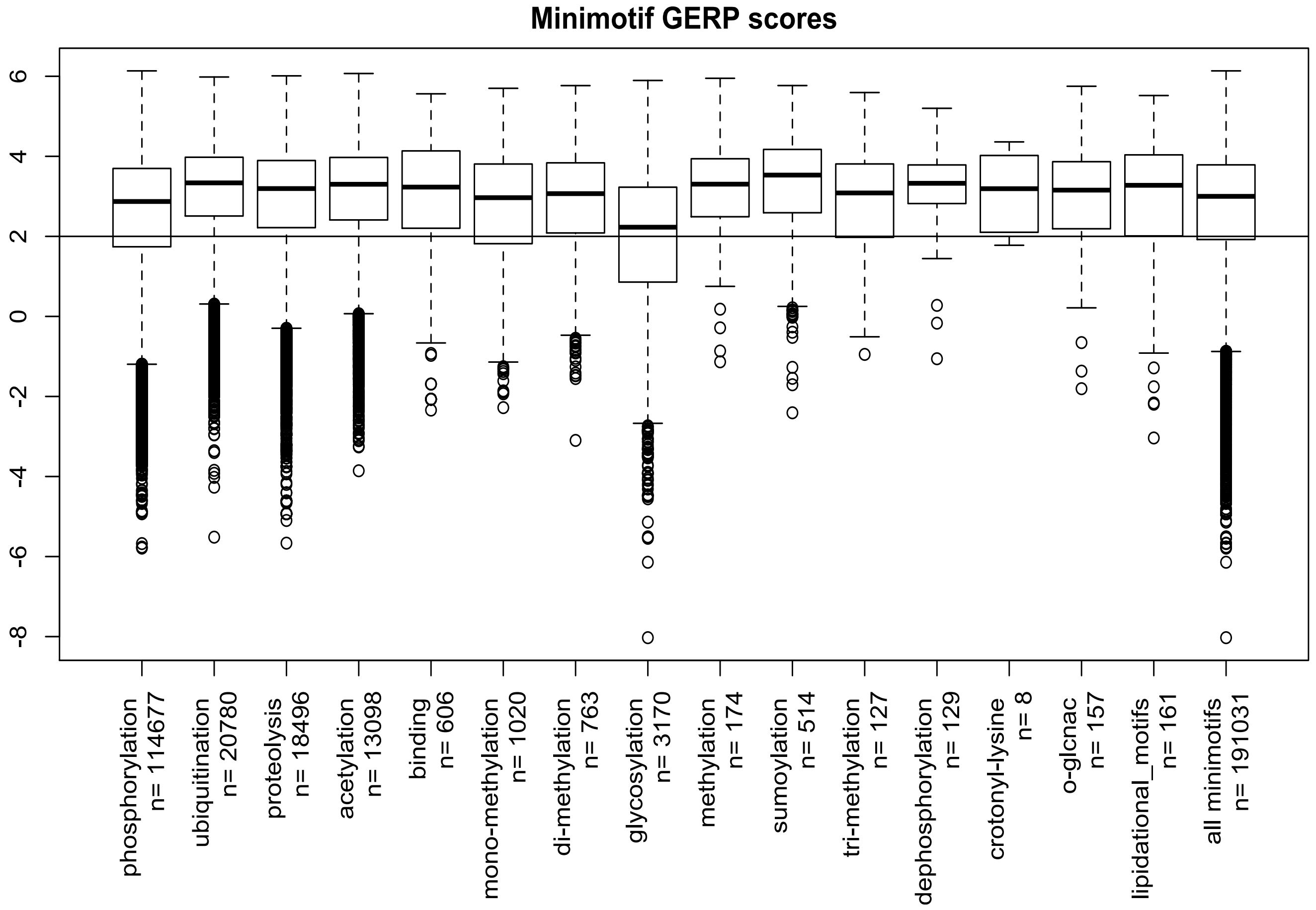

Figure S3. Quartile plots of GERP scores for different types of minimotif subactivities.

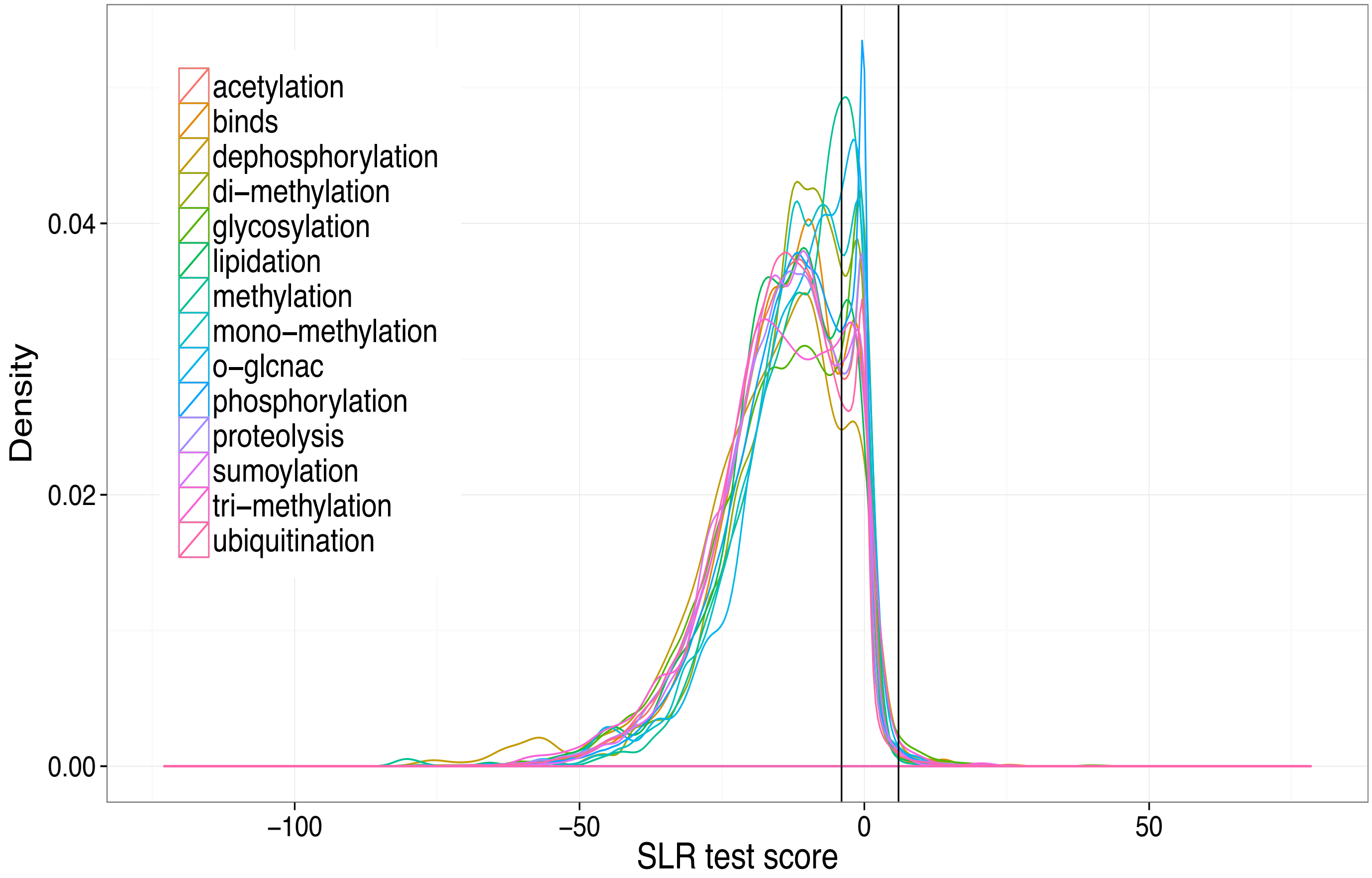

Figure S4. Density plot of SLR scores for different minimotif subactivities. Vertical lines indicated threshold for negative, neutral, and positive selection determined from **Fig. S2**.
